# Three‐year‐old Children Make Explicit Metacognitive Judgments – If Asked Appropriately

**DOI:** 10.1111/desc.70287

**Published:** 2026-09-30

**Authors:** Marlene M. Meyer, Marina Proft, Jan Engelmann, Hannes Rakoczy

**Affiliations:** ^1^ Department of Cognitive Developmental Psychology Georg‐August‐University Göttingen Göttingen Germany; ^2^ Department of Psychology University of California, Berkeley Berkeley California USA

**Keywords:** cognitive development, metacognition, metacognitive judgments, social context, uncertainty

## Abstract

Metacognition—the capacity to represent and reflect upon our knowledge and its sources—is fundamental for higher cognition and learning. Developmental research suggests that the ability to make explicit metacognitive judgments emerges surprisingly late, not before school age. However, this research has assumed that metacognition is optimized for private reflection and therefore tested children's metacognitive judgments in individual settings. In contrast, recent theoretical approaches propose that explicit metacognition may have a primarily social function and should therefore become most evident in socio‐communicative contexts. We tested this proposal across four pre‐registered studies (*N* = 195). First, we conducted two studies in which 3‐ and 5‐year‐old children participated in a social version of a well‐established metacognition task (*partial ignorance task*). Children had to communicate their uncertainty towards a cooperative partner who relied on their advice. Even 3‐year‐olds provided accurate metacognitive judgments under these conditions: They spontaneously and explicitly reported uncertainty when they were partially ignorant, but not when they were knowledgeable. Two control studies yielded mixed evidence regarding the question whether the enhanced metacognitive performance in the first two studies was indeed mainly due to the socio‐communicative context rather than other factors. Overall, the current findings suggest that explicit metacognition manifests earlier in development than previously assumed and may be boosted in some—particularly socio‐communicative—contexts.

## Summary

- Previous research suggests that spontaneous, explicit metacognition may emerge surprisingly late (around age 6).
- The present studies tested whether children reveal earlier metacognitive competence in an alternative, socio‐communicative test context.
- Here, children demonstrated explicit metacognition much earlier (from age 3): They provided accurate judgments about their own ignorance when communicating with an interlocutor.
- These findings highlight pragmatic relevance of metacognitive tests and support theoretical accounts which propose that explicit metacognition is primarily for social and communicative usage.

## 1 Introduction

Metacognition, commonly understood as cognition about cognition (Flavell 1979; Nelson and Narens 1994), is foundational for mature human thought. Knowing what we do or do not know, where we know something from and how certain we are, is crucial for deciding and planning how to seek, acquire, and manage information. Thus, the ability to monitor and control our ongoing cognitive processes makes it a key prerequisite for critical thinking. In light of this practical relevance, it is surprising how relatively late metacognition appears to develop in ontogeny.

### 1.1 Early Metacognition

Precursors of metacognition are present in early development and across species. For example, 2–to 3‐year‐old children as well as non‐human primates engage in procedural metacognitive monitoring and control: They keep track of when they lack relevant information (*monitoring*) and then, in response (*control*), engage in information seeking behavior (Call and Carpenter 2001; Kornell et al. 2007; Kloo et al. 2017; Lapidow et al. 2022) or opt‐out from risking uncertain choices (Balcomb and Gerken 2008; Kim et al. 2016; Lyons and Ghetti 2013; Templer and Hampton 2012). Further lines of research on uncertainty monitoring and control have demonstrated that toddlers and even infants selectively ask for help (Goupil et al. 2016; Coughlin et al. 2015), prefer certain over uncertain responses to be evaluated by another person (Hembacher and Ghetti 2014) and produce uncertainty gestures (Goupil and Kouider 2016; Kim et al. 2016).

These findings are impressive evidence for early metacognitive sensitivity in the widest sense: Infants and non‐human animals procedurally track and differentially respond to their own epistemic states (Proust 2013; Goupil and Kouider 2019; Goupil and Proust 2023). However, such procedural sensitivity (e.g., searching more when one does not know where an object is) falls short of fully‐fledged explicit metarepresentational metacognition: The capacity not only to differentially respond to uncertainty in the environment, but to monitor one's own epistemic states and explicitly meta‐represent them (“I do not know where the object is”). The differences between the former and the latter have been variously described as *procedural* versus *declarative*, *implicit* versus *explicit* or *model‐free* versus *model‐based* metacognition (Goupil and Kouider 2019; Perner 2012; Shea et al. 2014; Carruthers 2021; Carruthers and Williams 2022). Several dual‐process accounts have spelled out these differences in terms of *Type I* (procedural, implicit) versus *Type II* (declarative, explicit) metacognition (e.g., Goupil and Kouider 2019; Shea et al. 2014).

### 1.2 Later Developing Explicit Metacognition

Existing research suggests that declarative, explicit forms of metacognition develop in surprisingly protracted ways and only begin to emerge around the age of 5–6 years (Beran et al. 2012; Roebers 2017). Preschool children younger than this demonstrate a striking absence of metacognitive competence in various domains: For instance, a large body of evidence on meta‐memory reveals that young children tend to overestimate their knowledge and learning progress: Children claim to have understood instructions that are in fact false, incomplete or simply senseless (Markman 1977), or to have always known words they have only recently learned (Taylor et al. 1994). In various memory‐recall tasks, preschoolers persistently fail to correctly assess their learning progress and overconfidently predict better memory performance (Finn and Metcalfe 2014; Lipko et al. 2009).

Perhaps the most surprising and compelling evidence pertains to children's explicit self‐ascription of (un‐)certainty. When confronted with a hiding event, children have no difficulty to self‐ascribe knowledge in full knowledge conditions where they see the object being hidden and, analogously, to self‐ascribe ignorance in full ignorance conditions where they are told that some object has been hidden but have no clue what it might be (e.g., Rohwer et al. 2012). However, they fail to provide accurate metacognitive reports in crucial *partial ignorance conditions*. The only difference here (compared to an instance of full ignorance) is that children know the set of possible objects beforehand—they see that one of several objects (e.g., A, B, C) is placed in a container, but not which one. Strikingly, when now asked whether they know what is in the container, children up to 6 years provide inaccurate metacognitive judgments: They incorrectly state that they know and commit themselves to corresponding claims (e.g., “A is in the box”) with certainty and conviction (e.g., Rohwer et al. 2012; Kim et al. 2016; Kloo et al. 2017). This line of research thus suggests that even the most basic forms of declarative, explicit (Type II) metacognition develop slowly and in protracted ways well into the school‐age years.

### 1.3 Performance Rather Than Competence Limitations?

However, it may be that young children's difficulty with explicit metacognition tasks do not reflect lack of cognitive competence, but merely linguistic performance limitations. Such performance limitations may be lexical: Young children may be able to make explicit metacognitive judgments, but may be unable to put them into the corresponding words. Evidence for such a possibility comes from work by Ghetti and colleagues (for an overview, see Ghetti et al. 2013). Methodologically, this work removes lexical performance factors by allowing children to express uncertainty through pointing at simple pictorial scales. The 3‐point scales often used in these studies depict a person that looks either very confident, neutral or very unconfident. In some of the studies’ key conditions, children are confronted with blurry images in a picture identification task. After training with the scales, children aged 3 to 5 years point more towards the unconfident scale item on trials on which they give incorrect answers. These studies are very impressive in documenting previously masked early competence. Yet so far, they have uncovered proficiency in 3‐year‐olds only after some training (e.g., Lyons and Ghetti 2011, 2013; Hembacher and Ghetti 2014; Coughlin et al. 2015) or after supplying them with targeted previous metacognitive experiences (Lapidow et al. 2022). To date, there is no evidence for spontaneous explicit metacognitive judgments in 3‐year‐olds, expressed either lexically or by alternative symbolic means.

Another possibility is that previous research has underestimated early competence not (only) due to lexical, but due to pragmatic performance factors. The way children have been asked to make their metacognitive judgments may have been pragmatically confusing: An experimenter who controls and perfectly knows about the experimental situations asks the child about her epistemic status. The question is not a regular and sincere question (the experimenter does not lack information she asks for), but an academic test question. Thus, the child's expression of her metacognitive judgment has little pragmatic point—except to show that she understands the question and can play the experimental game. This may make the task artificial and difficult (for similar arguments and findings in Theory of Mind research, see e.g., Siegal and Beattie 1991; Helming et al. 2016; Westra and Carruthers 2017; Oktay‐Gür and Rakoczy 2017). It may be that metacognitive competence is already present, ready for use when really needed, but substantially masked in such pragmatically infelicitous situations. This fits with theoretical accounts which argue that the primary use of explicit metacognition is social and communicative.

### 1.4 Social Metacognition

Conceptually, most previous research builds on the assumption that the core function of metacognition is to promote individual reflection and cognition. This assumption has been challenged by recent theoretical alternatives. In line with general accounts that emphasize the social origins of higher human cognition (such as reasoning, argumentation or episodic memory, Tomasello 1999; Mercier and Sperber 2011; Mahr and Csibra 2018), the guiding idea is the following: The fundamental function of explicit metacognition is social. Its primordial form is communicating about one's knowledge and (un‐)certainty in joint coordination and problem‐solving, rather than reflecting on one's knowledge in isolation (Frith 2012; Nagel 2015; Shea et al. 2014). Sophisticated metacognitive skills should therefore be most relevant and most easily detectable in social tasks that require individuals to communicate, coordinate and cooperate with others (Shea et al. 2014). In support of this, several studies with adults have emphasized the crucial role of metacognitive communication in joint decision‐making. These studies have shown that exchanging and discussing individual degrees of certainty resulted in collective benefit and enhanced the quality of collective over individual decisions (Bahrami et al., 2012; Brennan and Enns 2015; Fusaroli et al. 2012; Schulz‐Hardt et al. 2006).

### 1.5 Rationale of the Present Study

Read ontogenetically, this social framework implies that when searching for the developmental origins of explicit metacognition, one should focus on socio‐communicative tasks. In previous metacognitive tasks (such as the partial ignorance tasks), children may have failed because they were asked to reflect on their knowledge privately; perhaps those tasks did not assess explicit metacognition in the right—that is, pragmatically meaningful and sufficiently social—context. One previous experimental study provides preliminary support for this view. Kim et al. (2016) showed that 3‐ and 4‐year‐old children performed significantly better in a partial ignorance task when they were asked to either inform or refrain from informing another agent about the identity of the hidden object instead of providing an explicit metacognitive judgment themselves. They informed this other agent significantly less often and produced more uncertainty gestures when partially ignorant. However, and in crucial contrast, the same children did not provide accurate explicit reports: When the experimenter explicitly asked whether they knew the hidden identity (now in an academic test question just like in the standard partial ignorance task; e.g., Rohwer et al. 2012), participants made overconfident judgments and incorrectly claimed to know which object was hidden (Kim et al. 2016). Thus, improved performance was only observed for implicit measures of metacognition within a socio‐communicative context. The effect of a social context on explicit measures (self‐ascription of (un‐)certainty), which are our focus here, has not been tested yet.

Therefore, the rationale of the present studies was to test the ontogenetic predictions of the social account of metacognition more directly. To this end, we developed an explicit partial ignorance task that is inherently social and communicative: The child participant interacts with a partner who lacks crucial information about the contents of a container, asks the child for their advice, and then relies on this advice to solve the task. The children themselves have either full knowledge (*full knowledge control condition*) or only partial knowledge (*partial ignorance test condition*) about the relevant state of affairs. In this way, and in crucial contrast to previous individual task settings as well as the social but implicit measure by Kim et al. (2016), we confronted children with a genuine metacognitive question that was embedded in a social context. In an initial proof‐of‐concept study (Study 1a), we investigated whether 3‐ and 5‐year‐old children—within suitably social task contexts—express uncertainty more often when partially ignorant than when knowledgeable; the findings document such metacognitive sensitivity in both age groups. Study 1b extends this initial evidence and addresses our central research question by testing whether children also *explicitly* self‐ascribe ignorance when directly asked. Studies 2 and 3 are follow‐up control studies to rule out that these effects of enhanced metacognitive judgments are due to potentially confounded factors other than the socio‐communicative context. All studies were preregistered (see supporting information including open access data and analyses on OSF: https://osf.io/ert43/?view_only=445f61d5252d402eb7c4cdb0f76c10d0).

## 2 Study 1a

Study 1a tested 3– and 5‐year‐old children's ability to communicate their uncertainty to a cooperative partner in a social paradigm of a partial ignorance and full knowledge task. This first study constitutes an initial proof‐of‐concept that children were sensitive to the presence of uncertainty in the current task design.

## 3 Methods

### 3.1 Participants

The final sample included a total of 66 typically developing German children aged 3 years (*n* = 34, 18 girls, *range* = 36–47 months*, M_months_* = 41.82, *SD_months_* = 3.29) and 5 years (*n* = 32, 14 girls, *range* = 60–71 months, *M_months_* = 65.09, *SD_months_* = 3.90). For all four studies, participants were recruited from a local database of German‐speaking children whose parents had previously expressed interest in child development studies. The participating children lived in a mid‐sized German university city and its surroundings. Further demographic data were not collected due to the local data protection rules (see Singh et al. 2024). Sample sizes were preregistered based on those from comparable previous studies (e.g., Kim et al. 2016). Ten additional children were tested but excluded from analyses because they neither communicated (*n* = 2) nor cooperated (*n* = 4) with the experimenters, aborted the session (*n* = 1) or because their caregivers interfered (*n* = 3). Study 1a was primarily conducted in the department's facilities (*n* = 40), as well as in local kindergartens (*n* = 26) by two female experimenters (main: E1; and children's partner: E2).

### 3.2 Design and Procedure

We adopted a within‐subjects design: Children participated in two conditions in counterbalanced order, with two consecutive trials per condition (four trials in total): Our conditions were adapted from a standard partial ignorance (test condition) and respective full knowledge task (control condition; Rohwer et al. 2012). Crucially, the metacognitive tasks were embedded in an inherently socio‐communicative context within the interaction between the child participant and E2. A communicative and cooperative relationship between them was established before the actual task commenced. In this warm‐up, all materials were introduced, and children learned that it was acceptable to acknowledge uncertainty or ignorance. Furthermore, E1 established the mutual understanding that children and E2 could help each other. See S1 for detailed description of the procedure and materials.

The general task structure of the test phase was as follows: In each of the four trials (two trials per condition), E1 hid one out of three previously exposed toy animals inside a three‐sided box. E2 was instructed to identify the hidden animal to receive a reward. In absence of E1, E2 then turned to the child who sat, depending on condition, in front of a transparent window (control condition: full knowledge) or blurry window (test condition: partial ignorance), and asked the metacognitive test question (see Figure 1). In Study 1a, we aimed at eliciting children's spontaneous metacognitive responses as naturally as possible and thus, asked an open test question (“Can you help me? Can you tell me which animal it is?”). If children replied without explicitly referring to their epistemic states (e.g., when only offering advice “The lion!”), E2 posed a follow‐up question (“Ah, you think it is the lion—are you sure?”). If children only affirmed or negated the initial test question, E2 respectively followed up by asking “And which animal is it?” or by accepting their ignorance statement (“Okay, then I guess on my own”). Eventually, E2 asked E1 to return and then committed to an answer (always adopting children's advice if they provided any), and E1 revealed the hidden animal. E2 was rewarded with a sticker only if her answer was correct. Children did not receive any stickers, but after they completed all test and control trials, E1 instructed them to switch roles so that it was them who now relied on E2's advice to earn stickers. These trials were not analyzed but served as upholding children's motivation and reciprocal helping behavior with E2.

**FIGURE 1:**
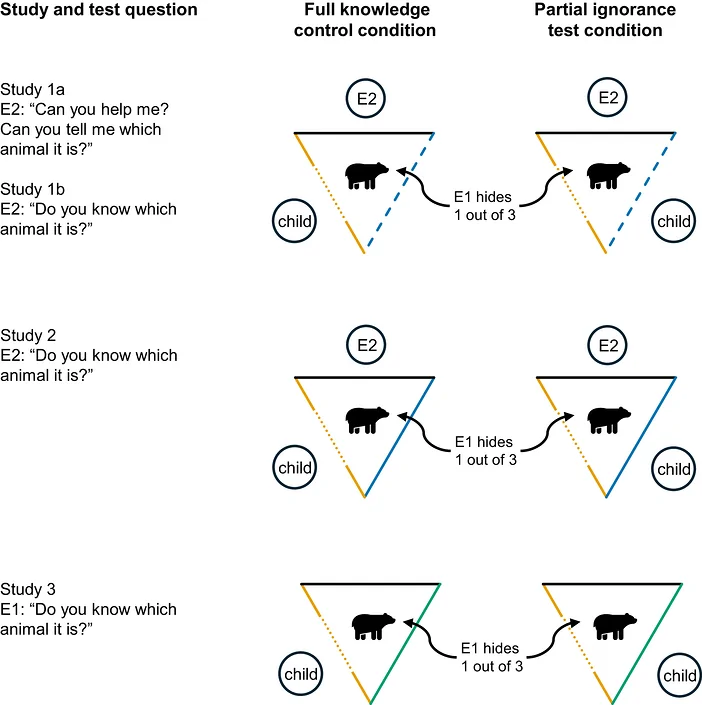
**Overview of conditions and test questions across studies**. *Note*: The experimenter (E1) secretly hid one out of three toy animals inside the three‐sided box. Test questions were asked by the ignorant partner (E2), with E1 being absent (Studies 1a‐ 2), or by E1 herself (Study 3). Solid lines represent opaque side. Dashed lines represent blurry window. Dotted orange lines represent transparent window. Children partook in two trials per condition, with counterbalanced order of conditions. Each animal was hidden at least once, the order of hidings and identity of animals hidden twice was randomized.

### 3.3 Coding

In Study 1a, the main dependent variable was a binary trial‐level indicator of whether children expressed uncertainty (0 = no, 1 = yes) in response to their partner's prompts (“Can you tell me which animal it is? … Are you sure?”). Because of the liberal nature of this first proof‐of‐concept study, we coded explicit ignorance acknowledgements (e.g., “I do not know”), as well as any other instance of expressing uncertainty verbally (e.g., “It is either the bear or the lion”, “Maybe the sheep?”, “I cannot see it”), nonverbally (e.g., uncertainty gestures, raising their voice at the end of guessing a specific animal (“Lion…?”)) and through other behavioral responses such as information‐seeking (e.g., attempting to peek through transparent window or to open the box). All sessions were videotaped and coded by E1. Reliability coding by a blinded coder for 25% of the videos revealed 100% agreement for children's uncertainty expressions.

#### 3.3.1 Results and Discussion

Following our preregistered analysis plan, we fitted binomial generalized linear mixed models (GLMMs) to investigate the effects of our main predictors condition, age group, the interaction between condition and age group, order of condition, as well as the random effect of participant ID on children's likelihood to spontaneously express uncertainty. The full model fitted the data significantly better compared to a null model that lacked all main predictors but included the same random effect structure (*X* ^2^ = 139.17, *df* = 4, *p* < 0.001; see S1for detailed information). However, the interaction between condition and age group was not significant (*b* = 0.77, *p* = 0.38) so we fitted a reduced model without the interaction term. This model revealed a significant effect of condition (*b* = 4.60, *p* < 0.001), but no significant effects for age group (*b* = 0.34, *p* = 0.54) or order of condition (*b* = 0.74, *p* = 0.18).

Thus, children in both age groups demonstrated metacognitive sensitivity by consistently expressing more uncertainty in the partial ignorance than in the full knowledge condition (Figure 2). Both 3‐ and 5‐year‐olds frequently expressed uncertainty under partial ignorance but rarely when they had full knowledge (see Table 1 for observed probabilities, model‐fitted values and corresponding confidence intervals).

**FIGURE 2:**
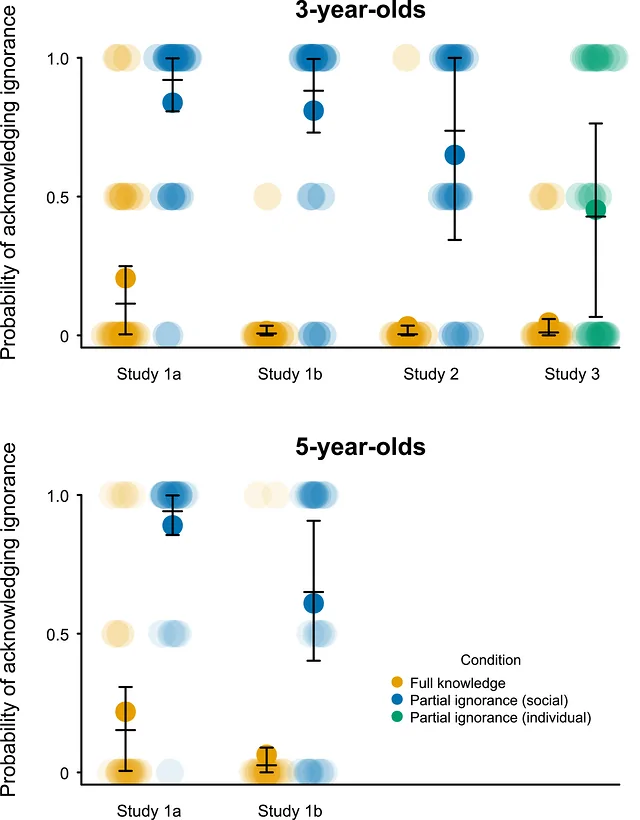
**Children's observed proportion and predicted probability of acknowledging ignorance as a function of condition, age and study**. *Note*: Semi‐transparent points represent individual children's proportion of acknowledging ignorance across trials (maximum two trials per condition); solid points represent mean proportion of acknowledging ignorance aggregated across participants. Horizontal line segments with error bars depict the fitted model values and their 95% confidence limits obtained from 1000 bootstraps.

**TABLE 1:** Probabilities of acknowledging ignorance across studies and conditions.

					95% CI	
	Condition	Age group	Observed mean	Fitted value	LL	UL
Study 1a	Full knowledge	3	0.206	0.114	0.004	0.249
		5	0.219	0.153	0.005	0.308
	Partial ignorance	3	0.838	0.920	0.807	0.998
		5	0.891	0.942	0.856	0.999
Study 1b	Full knowledge	3	0.016	0.007	0	0.035
		5	0.064	0.026	0	0.089
	Partial ignorance	3	0.810	0.881	0.730	0.996
		5	0.609	0.650	0.403	0.907
Study 2	Full knowledge	3	0.031	0.004	0	0.035
	Partial ignorance	3	0.629	0.704	0.345	1
Study 3	Full knowledge	3	0.046	0.011	0	0.059
	Partial ignorance	3	0.453	0.428	0.066	0.763

*Note*: Observed means correspond to observed mean proportion of trials in which children acknowledged their ignorance (maximum two trials per condition) aggregated across participants; fitted values and 95% CIs were derived from individual studies’ model and respective bootstraps (*n* = 1000); CI = confidence interval; LL = lower limit; UL = upper limit.

The finding that already 3‐year‐old children's uncertainty judgments were boosted in our social version (compared to standard partial ignorance tasks) provides initial evidence for the claim that the primary function of explicit metacognition is social. However, given the very liberal coding of metacognitive performance in response to an open test question, we cannot distinguish truly *explicit* metacognition from more implicit forms in fully stringent ways. Study 1b, therefore, tested children's explicit metacognitive judgments with more stringent measures.

## 4 Study 1b

### 4.1 Methods

#### 4.1.1 Participants

Study 1b tested thirty‐two 3‐year‐olds (17 girls, *range* = 36–47 months, *M_months_* = 42.13, *SD_months_* = 3.17) and thirty‐two 5‐year‐olds (15 girls, *range* = 60–71 months, *M_months_* = 66.41, *SD_months_* = 3.04). Five additional children were tested but retained from analyses because they did not communicate with the experimenters (*n* = 1), did not complete the session (*n* = 1), or because of experimental error (*n* = 1) or parental interference (*n* = 2). All sessions were conducted in the department's facilities by two female experimenters (E1 and E2), videotaped and coded by one of the experimenters. Again, reliability coding for 25% of the videos revealed perfect agreement between coders (100%).

#### 4.1.2 Design and Procedure

The main experimental design and procedure was the same as in Study 1a with the following crucial difference: The test questions were rephrased to explicitly refer to children's epistemic state and avoid ambiguous responses (as E2 combined two questions in Study 1 “Can you help me? Can you tell me (…)?” which made interpretation of children's answers difficult). When E2 turned to the children for help, she asked a single precise metacognitive question (“Do you know which animal it is?”, see Figure 1). If children responded without referring to the crucial state of knowledge (e.g., suggesting “Lion!” without indicating their epistemic state), E2 followed up to obtain a definite metacognitive answer (“Do you know this?”). Additionally, the coding scheme was stricter compared to coding in Study 1a and only truly explicit metacognitive judgments were counted. In this way, Study 1b is more directly comparable with previous non‐social partial ignorance tasks (Rohwer et al. 2012; Kim et al. 2016). Moreover, we removed a potential confound in E2's wording (e.g., when asking for help and when giving her final answer to E1): In contrast to Study 1a, E2 refrained from using the verb *to guess* (German: “raten”) but used a more neutral phrasing *(to say*, German: “sagen”) in Study 1b (e.g., “Okay, I say it is the sheep, then”).

#### 4.1.3 Coding

In Study 1b, children's metacognitive competence was coded more conservatively with regards to whether they *explicitly* acknowledged their ignorance (1 = yes, 0 = no). To this end, children's responses were only considered indicative of metacognitive competence if they explicitly negated the main test question (e.g., “No, I don't know”, head shaking) or if they acknowledged that their initial response was a guess by negating the follow‐up question (e.g., “Maybe the lion”—“Do you know this?”—“No”). Note that nonverbal negation responses like head shaking were sufficient, whereas implicit markers of children's uncertainty, such as shoulder shrugging or information‐seeking behavior, were not coded as acknowledging ignorance.

### 4.2 Results

Following preregistered analysis plans, we fitted a GLMM that included the fixed effects of condition, age group, the interaction of condition and age group, as well as random intercepts for individual participants. We found a significantly better fit of this full model as compared to its respective null model (*X* ^2^ = 165.68, *df* = 4, *p* < 0.001). Because the interaction between condition and age group was significant (*b* = −2.89, *p* = 0.02), we ran separate analyses in both age groups. For each age group, we fitted a GLMM to test the fixed effects of condition, order of condition and random intercepts for individual children on their probability to acknowledge ignorance. Model comparisons yielded significantly better fits of the respective full models (*p*s < 0.001) which revealed significant effects of condition (Figure 2): For the 3‐year‐olds, the full model revealed a significant effect of condition (*b* = 7.02, *p* < 0.001), with high probabilities of acknowledging ignorance only in the partial ignorance condition (see Table 1), and no significant effect of order of condition (*b* = −1.27, *p* = 0.15). For the 5‐year‐olds, the full model revealed significant effects of condition (*b* = 4.86, *p* < 0.001, see Table 1) as well as of order of condition (*b* = −1.85, *p* = 0.04).

Hence, 3‐year‐old children explicitly acknowledged their ignorance significantly more often under partial ignorance than when fully knowledgeable, independently of which condition was presented first. The effect of condition on children's self‐ascription of ignorance was similar but less pronounced in 5‐year‐old children, as they also acknowledged their ignorance significantly more often in the partial ignorance than full knowledge condition but tended to do so more when they experienced the full knowledge condition first.

### 4.3 Discussion

The results from Study 1b converge with those from Study 1a: Although 3‐ and 5‐year‐old children were tested with a more conservative test question and a stricter, corresponding coding scheme, findings confirmed that both age groups reveal explicit metacognition in a social partial ignorance task. This speaks against the concern that different phrasing of the test questions and coding of uncertainty can account for the competent metacognitive performance observed in Study 1a.

Taken together, Studies 1a and 1b indicate that children as young as 3 years of age competently engage in spontaneous, explicit metacognition—once the task context has a suitable socio‐communicative structure: When their partner asked them genuine metacognitive questions, participants correctly claimed to know when they had access to the respective information, but correctly acknowledged their uncertainty when they were partially ignorant. This stands in stark contrast with previous findings that when children are asked to reflect on their partial ignorance in individual task contexts, they fail to provide explicit metacognitive judgments until much later. Studies 1a and 1b thus converge in indicating that children reveal earlier explicit metacognition in the social tasks here compared to non‐social tasks of previous studies. They thus support the general idea that guides the present research: That the prime function of explicit metacognition is social; and that therefore the primary domain in which metacognitive competence emerges and reveals itself is social interaction, communication and cooperation (Shea et al. 2014).

However, from Studies 1a and 1b alone it remains somewhat unclear whether indeed the relevant difference in performance between the present and previous tasks reflects the influence of socio‐communicative versus individualistic task structures, or whether there may be alternative explanations of the different findings. Consistent with pragmatic modifications of the task, one potential alternative explanation concerns a detail of our methodology.

In previous partial ignorance tasks, one of several pre‐exposed objects was hidden inside an opaque container (e.g., Rohwer et al. 2012; Kim et al. 2016; Kloo et al. 2017). In contrast, in the present studies, the object was hidden inside a container with a blurry window. Therefore, children might have dealt with *perceptual* uncertainty rather than *epistemic* uncertainty. The blurry window might have served as a visual reminder of their uncertainty throughout partial ignorance test trials, reducing demands for children's working memory and thereby making it easier for them to express uncertainty. Whereas the standard partial ignorance task requires participants to maintain and reflect on mental representations, the blurriness could directly be perceived and therefore judged more easily (Harris 1995). We address this alternative explanation in Study 2.

Similarly, we might have enhanced children's performance by altering other factors of the standard procedure. For instance, children might have been more engaged with the three‐sided box providing a constant contrast between knowable and unknowable trials, or might have felt more motivated by the possibility to collect rewards. In order to argue for a primarily social function and boost of explicit metacognition, a minimal contrast between social and non‐social conditions is needed. We address this point in Study 3.

Since we assumed that these potential issues would affect performance in 3‐ and 5‐year‐old children equally, and since we were interested in testing whether *earlier emerging* forms of explicit metacognition are boosted by social contexts, we decided to test only the younger age group in Studies 2 and 3.

## 5 Study 2

### 5.1 Methods

#### 5.1.1 Participants

Study 2 includes data of 32 3‐year‐olds (15 girls, *range* = 36–47 months, M*_months_* = 41.62, SD*_months_* = 2.77). Seven additional children were tested but excluded from analyses because they did not communicate with the experimenters (*n* = 1), did not complete the session (*n* = 4), or had language difficulties (*n* = 2). Again, all participants were tested in the department's facilities by two female experimenters (E1 and E2), all sessions were videotaped and coded by one of the experimenters (reliability coding for 36% of the videos revealed 100% agreement with a second coder).

#### 5.1.2 Procedure and Coding

Study 2 controlled for the alternative explanation that children were visually reminded of their uncertainty in Studies 1a and 1b rather than providing evidence for social metacognition. Thus, E1 and E2 followed the same procedure and applied identical coding as in Study 1b. The only but crucial difference was that the three‐sided box did not involve a visual representation of children's uncertainty—that is, the partial ignorance condition took place in front of a completely opaque side instead of a blurry window (see Figure 1).

### 5.2 Results and Discussion

Analyses were conducted as preregistered (detailed information in S1). The converging model (GLMM) included condition and order of condition as fixed effects, and random intercepts for individual participants. It fitted the data significantly better than the respective null model (*X* ^2^ = 73.39, *df* = 2, *p* < 0.001) and revealed significant effects of condition (*b* = 7.47, *p* < 0.001) and order of condition (*b* = −2.49, *p* = 0.04) on children's likelihood to acknowledge ignorance. Thus, Study 2 replicated the main findings from Studies 1a and 1b: Three‐year‐old children competently declared being ignorant more often in the partial ignorance than in the full knowledge condition. In Study 2, they did so more often when the partial ignorance condition was presented secondly.

Although children's probability of acknowledging ignorance was high under partial ignorance and low under full knowledge, they competently acknowledged their ignorance descriptively less often than in Study 1b (see Table 1, Figure 2). Therefore, we conducted exploratory analyses across the 3‐year‐old children from Studies 1b and 2 to directly assess the influence of the blurry versus no window on children's metacognitive reports. We fitted a GLMM including the fixed effects of condition, study, order of condition, their potential interactions and random intercept for participant ID. The model did not converge before excluding the three‐way interaction of condition, study and order of condition. As the remaining interaction between condition and study was not significant (*b* = −1.62, *p* = 0.32), it was removed. This reduced model (including condition, study ID and order of condition as fixed effects and random intercepts for participant ID) confirmed that condition was a significant predictor of children's ignorance statements (*b* = 7.55, *p* < 0.001) as well as the order of condition (*b* = −1.86, *p* = 0.02), but study was not (*b* = −1.23, *p* = 0.10). This means that 3‐year‐olds’ explicit metacognitive judgments in the partial ignorance condition did not depend on whether they sat in front of a blurry window (Study 1b) or at the opaque side of the box (Study 2).

Thus, Study 2 controlled for the alternative explanation that our paradigm was confounded with children's ability to reflect on perceptual rather than epistemic uncertainty. The blurry window might have helped 3‐year‐old children (descriptively more accurate ignorance judgments in Study 1b), but their competence cannot be explained away by the presence of such a visual reminder of their uncertainty. Rather, Study 2 provides further evidence that explicit metacognition is boosted in socio‐communicative contexts. Still, however, a minimal contrast between closely matched social and non‐social conditions is needed.

## 6 Study 3

Study 3 implements a non‐social test condition closely matched to the social conditions used so far to enable minimal contrast comparisons.

### 6.1 Methods

#### 6.1.1 Participants

Thirty‐three 3‐year‐old children (16 girls, *range* = 36–47 months, *M_months_* = 40.24, *SD_months_* = 3.28) participated in Study 3. Data of one additional child was dropped from analyses because of profound language difficulties. All sessions were videotaped; reliability coding for 25% of the videos revealed 100% agreement between two independent coders.

#### 6.1.2 Procedure and Coding

Since a direct comparison of our social partial ignorance task (Studies 1b and 2) with a non‐social control group was needed, the children were tested by the same main experimenter (E1) with the same materials and experimental setup as in Study 2 – the crucial difference was the absence of a cooperative partner and thus the social context. Children were introduced to the three‐sided box and overall game just as in Studies 1b and 2, but in the test phase, E1 was posing the metacognitive test questions after hiding one of the toy animals inside the box herself (see Figure 1). Children's responses were coded with regards to whether they acknowledged their ignorance as conservatively as in Studies 1b and 2.

### 6.2 Results and Discussion

The model (GLMM) that fitted the data significantly better than the respective null model (*X* ^2^ = 40.72, *df* = 2, *p* < 0.001) included the fixed effects of condition, order of condition and random intercept for participant ID. It revealed significant effects on children's probability to acknowledge their ignorance for condition (*b* = 4.32, *p* < 0.001), not for order of condition (*b* = 0.77, *p* = 0.43): Three‐year‐old children in our non‐social task acknowledged their ignorance significantly more under partial ignorance than in the full knowledge control condition (see Table 1).

However, descriptively, children seemed to perform more poorly in the non‐social control task (Study 3) than in the social variants (Study 1b and 2). Therefore, we conducted exploratory control analyses on the performance of the 3‐year‐olds across Studies 2 and 3 (the studies most closely matched, in a minimal contrast, in all respects except the social vs. individual task format). We specifically tested whether there was a significant interaction between study and condition—this would demonstrate that children performed better on the social relative to the non‐social version of the task. We fitted a GLMM including the effects of condition, the interaction between study and condition, as well as order of condition and random intercepts for participants. This model had a significantly better fit than the respective null model (*X* ^2^ = 112.84, *df* = 4, *p* < 0.001) and revealed no significant interaction between study and condition (*b* = −2.22, *p* = 0.11) and no effect for order of condition (*b* = −0.68, *p* = 0.38), indicating that the effect of condition only tended to be stronger in Study 2, that is, in the social compared to the non‐social task.

Thus, the statistical difference between studies is less substantial than expected, showing that children were metacognitively competent in both social and non‐social contexts. Descriptively though, the overall effect of condition tended to be stronger in Study 2 than in Study 3 so that children appeared to acknowledge their partial ignorance more in the social compared to the non‐social task. The conjecture that these descriptive trends are meaningful is further corroborated by comparing the mean proportions of partial ignorance trials in which 3‐year‐old children acknowledged their ignorance between the present studies and previous research: In the present non‐social task (Study 3), 3‐year‐olds provided accurate ignorance statements in about 43% of partial ignorance trials, compared to the 22% and 30% observed by Rohwer and colleagues (2012; Experiment 1) and Kim et al. (2016), respectively. Crucially, however, this is notably less than the 81% and 63% observed in *social* partial ignorance trials from the present Studies 1b and 2, respectively (see Table 1). Therefore, the boost in explicit metacognition that we observe here in Studies 1a, 1b and 2 relative to previous studies likely goes beyond what could be explained by non‐social modifications of the task alone.

Still, the unexpectedly improved performance in Study 3 compared to previous non‐social conditions warrants further consideration of how the present task modifications may have scaffolded young children's understanding of their uncertainty. Perhaps our design provided a clearer contrast between certain and uncertain trials and thus made the task more intuitive than previous studies. Additionally, the experimenter‐child interaction—while non‐cooperative—might have been “social enough” to enhance the relevance of the task and encourage metacognitive communication^1^. These possibilities raise important open questions for further research (see General Discussion).

## 7 Exploratory Analyses Across Studies 1b, 2 and 3

Although we find explicit metacognition in children as young as 3 years of age and provide some evidence in favor of the prediction that explicit competence may be particularly boosted in socio‐communicative contexts, one potential concern with the present findings is the following: Perhaps children's performance in response to repeated metacognitive questions does not necessarily reflect metacognitive insight. Rather, children may take pragmatic shortcuts to arrive at the correct answer without really engaging in metacognitive self‐ascription. In particular, maybe they merely respond to repeated questioning and take this as an indicator that their previous answers were wrong (e.g., Siegal et al. 1988; Bonawitz et al. 2020). If this was the case, one would expect to frequently see the following pattern across seemingly competent children: Initially, children do not show any metacognitive sensitivity. When asked “Do you know what is in the box?” in partial ignorance conditions, they would say, for example, “It is the lion”; but when then asked “The lion. So, do you know this?” they would pragmatically get the impression that something with their previous answers must have been wrong and switch to saying “No”.

To test for such pragmatic artifacts, we analyzed children's response patterns: When children finally gave competent answers (“No/ I do not know”), did they do so spontaneously (already in response to the first questions) or only after repeated questioning? The results were very clear: The majority of 3‐year‐olds across contexts spontaneously admitted that they did not know which animal was hidden inside the box already in response to the initial test question. In the social context (Studies 1b and 2), around 90% of competent children spontaneously acknowledged their ignorance; only few children initially guessed an animal but then revised their answer (*n* = 5 trials) and even fewer admitted to their ignorance after initially claiming to know (*n* = 3 trials). As revealed by Fisher's Exact Test, this pattern was not significantly different in the non‐social context (Study 3, *p* = .09), where around 70% of competent ignorance statements were made spontaneously. Thus, it is unlikely that children's expression of uncertainty across studies merely reflects a pragmatic artifact.

## 8 General Discussion

Across four studies, we found that even 3‐year‐old children are capable of engaging in explicit metacognition in a partial ignorance task. Especially in rich socio‐communicative task contexts, children appropriately indicated their uncertainty towards a partner when they themselves were partially ignorant, but did not do so when fully knowledgeable. Hence, the present set of studies provides further evidence that explicit metacognitive competence develops earlier than previously assumed. More specifically, our main results stand in contrast with many previous findings that children consistently fail metacognitive partial ignorance tasks up until age 6 (Kim et al. 2016; Rohwer et al. 2012; Perner 2012). The present studies demonstrate that even 3‐year‐olds *spontaneously* provide *explicit* metacognitive judgements about partial information without relying on confidence scales. Therefore, the present results extend previous research in at least two important ways.

First, the current design taps children's *spontaneous* communication of their own epistemic states in natural social interactions. Previous studies by Ghetti and colleagues show that children from around age 3 to 4 can use pictorial scales to express (un‐)certainty once previously trained with the scale or primed with specific metacognitive experiences (Coughlin et al. 2015; Ghetti et al. 2013; Lyons and Ghetti 2011, 2013; Hembacher and Ghetti 2014; Lapidow et al. 2022). The present findings extend this line of research by indicating truly spontaneous proficiency that does not depend on prior training or priming.

Second, the present work builds on and extends initial evidence that young children's metacognitive sensitivity benefits from more social task contexts by Kim et al. (2016). In that study, however, 3‐ and 4‐year‐old children only indicated their own ignorance more competently with implicit measures, whereas they did not reveal any explicit metacognition: When it came to their explicit judgments about their own epistemic states, they persistently overestimated their knowledge. In the current study, in contrast, already 3‐year‐old children overcame this overconfidence and made explicit metacognitive reports.

Crucially, previous studies (including Kim et al. 2016) have typically tested for children's explicit metacognitive judgments in individual settings. In contrast, the main set of studies here (Studies 1a, 1b, 2) tested children's metacognitive judgments towards relevant others in socio‐communicative settings. Importantly, the boost that we observed relative to previous findings was unlikely to be reducible to purely superficial characteristics of the study materials (as revealed by methodological control Study 2). Further concerns to the effects that children might have solved the present task by pragmatic short‐cuts could be ruled out, too (as revealed by exploratory analyses). These results build on and extend previous findings with adults and are compatible with a social primacy account of metacognition (Frith 2012; Shea et al. 2014): Explicit metacognition may be first and foremost for social use in communication and coordination. Accordingly, the developmentally primary forms of metacognition may be social: Communicating about one's knowledge in situations where this matters for interpersonal coordination. On this picture, private metacognition—think of Descartes reflecting solitarily about the foundations of his individual knowledge—does exist, of course. It might be, however, a developmentally secondary phenomenon, derived from primarily social and interpersonal metacognition.

However, this hypothesis was not fully supported by the individual control Study 3. Although the metacognitive boost tended to be somewhat greater in the social as compared to the non‐social settings, more children explicitly acknowledged their ignorance in the current non‐social context than in previous studies (e.g., Rohwer et al. 2012; Kim et al. 2016). A conceivable interpretation would be that reducing general task demands already promotes young children's private metacognitive insight which may then be boosted further in socio‐communicative contexts. One candidate factor that might have reduced task demands in the current task design (thus also in non‐social Study 3) pertains to the three‐sided box. It is possible that the contrast between knowable and unknowable conditions was made more salient by the inherent representation of this contrast between a side with a clear window and a side with a blurry window (Studies 1a, 1b) or no window (Studies 2, 3). This explanation is in line with the finding by Lapidow et al. (2022) that first‐hand experiences with being wrong about an initially overconfident judgment supports children's ability to evaluate their own uncertainty in subsequent trials. Both pieces of evidence suggest that the development of explicit metacognition may not only depend on socio‐pragmatic factors of the context, but that other factors like experiencing disconfirming evidence and surprise (as in Lapidow et al. 2022) or salient contrasts between knowable and unknowable situations (as in the present studies) shape young children's evaluation of epistemic uncertainty.

Regarding future research, the present results thus raise a number of important questions. Methodologically, one relevant question is which specific characteristic of our experimental approach contributed to the improved performance of young children. First, in our social task, the child and their partner were interdependent in their cooperative and coordinative efforts. Second, the pragmatics of the communicative interaction was more natural and felicitous: Rather than asking an academic test question without practical relevance (as in previous research), the partner in our experimental setup was in need of real information and thus asked a genuine question. Future studies could disentangle these factors related to cooperation, coordination and communication and test for their (independent or joint) roles in boosting metacognition. Third, which non‐social modifications of our task may have affected children's metacognitive understanding? We have discussed how experiencing the direct contrast between knowable as opposed to unknowable conditions may promote an earlier understanding of uncertainty. Further studies are needed to differentiate more systematically between social and non‐social factors that affect young children's metacognition.

In broader theoretical perspective, one central question relates to how early, implicit metacognition develops into more sophisticated and flexible, explicit forms of cognitive control. Various dual process views have analyzed the contrasts, in terms of representational scope and limits, between basic, early, model‐free and implicit metacognition and later, full‐fledged, model‐based and explicit metacognition (Carruthers 2021; Goupil and Kouider 2019; Shea et al. 2014). But little is known so far about the developmental transition mechanisms: How do children move from the former to the latter? Which roles do language, maturation and social experience play, for example?

An even more fundamental question pertains to the various forms of developmental progression from primary social to secondary more private uses of higher cognition. Different lines of research have discussed such patterns in reasoning (Mercier and Sperber 2011), episodic memory (Mahr and Csibra 2018) or, like in the present case, metacognition (Frith 2012; Shea et al. 2014; Heyes et al. 2020). But how do developments in these different areas relate to each other? Following Vygotsky, one would assume a bigger pattern of developmental systematicity behind these different phenomena: Higher cognition emerges first in social context and use; and development then proceeds by internalization from interpersonal to intrapersonal forms of cognition (Tomasello 1999; Vygotsky 1978). Natural language may play a crucial role in this process. It may provide the means to transform or “re‐describe” (Karmiloff‐Smith 1992) more basic and implicit capacities (implicit inference, memory or metacognition) into fully‐fledged explicit ones (explicit reasoning, episodic memory or metacognition) over development. Such Vygotskian ideas are highly compatible with more recent versions of core knowledge accounts according to which natural language plays a crucial role in transforming domain‐specific core knowledge into fully‐fledged explicit conceptual capacities (Carey 2009; Spelke 2022; Xu 2019). Future studies should test for such bigger developmental patterns of potentially social origins of various forms of explicit higher cognition across domains.

## CRediT Authorship Contribution Statement

**Marlene M. Meyer**: Conceptualization, Data curation, Formal analysis, Investigation, Methodology, Project administration, Visualization, Writing—original draft, Writing—review & editing. **Marina Proft**: Conceptualization, Methodology, Project administration, Supervision, Writing—review & editing. **Jan Engelmann**: Conceptualization, Methodology, Project administration, Supervision, Writing—review & editing. **Hannes Rakoczy**: Conceptualization, Methodology, Resources, Supervision, Writing—original draft, Writing—review & editing.

## Funding

This work was supported by the Deutsche Forschungsgemeinschaft (DFG, German Research Foundation)—GRK2906 – project number 502807174; the Deutsche Forschungsgemeinschaft (DFG, German Research Foundation)—SFB 1528: Cognition of Interaction; and Deutsche Forschungsgemeinschaft (DFG, German Research Foundation)—Project number 254142454/GRK 2070.

## Ethics Approval Statement

This research was conducted in accordance with the Declaration of Helsinki and the Ethical Principles of the German Psychological Society (DGPs), the Association of German Professional Psychologists (BDP), and the American Psychological Association (APA). It involved no invasive or otherwise ethically problematic techniques and no deception (and therefore, according to National jurisdiction, did not require a separate vote by a local Institutional Review Board; see the regulations on freedom of research in the German Constitution (§ 5 (3)), and the German University Law (§ 22)).

## Conflicts of Interest

We declare no competing interests.

## Supporting information




**Supporting information**: desc70287‐supp‐0001‐SuppMat.docx

## Data Availability

All anonymized data, analysis script and preregistrations are available on OSF (https://osf.io/ert43/?view_only=445f61d5252d402eb7c4cdb0f76c10d0).

## Endnotes

1 See Hembacher & Ghetti (2014) for similar manipulations of the child‐experimenter interaction that were more socio‐pragmatically felicitous without being fully cooperative.
