# Supplementary material for: Three‐year‐old Children Make Explicit Metacognitive Judgments – If Asked Appropriately
Source: Dev Sci. 2026 Sep 30;29(6):e70287. doi: 10.1111/desc.70287 (PMC13625023; doi:10.1111/desc.70287)
Supplement: Supplementary file 1 [file DESC-29-e70287-s001.docx]

**Supplementary Materials:**

Three-year-old children make explicit metacognitive judgments – if asked appropriately

**Participants and exclusions**

Our final sample included a total of 195 children, 131 3-year-olds (32 per study, except 34 in Study 1a and 33 in Study 3) and 64 5-year-olds (across Studies 1a and 1b, 32 per study). Sample sizes were determined conventionally (matched to comparable experiments in the literature, e.g., Kim et al., 2016) and preregistered prior to data collection. The number of tested 3-year-old children in Studies 1a and 3 deviates from the preregistered sample size due to invitation error. *Before* looking at the data, we decided to include them as it would have been a waste of resources to exclude the already obtained data.

An additional 21 children were tested but excluded from analyses. Based on our preregistered drop-out criteria, we excluded children who did not complete the test session (*n* = 6) or if experimental error occurred (*n* = 1). The other 14 children were excluded because their caregivers interfered during test session (*n* = 3), or because they neither cooperated (*n* = 2) nor communicated (*n* = 4) with the experimenters, partly because of language difficulties (*n* = 3). Unfortunately, we missed to explicitly preregister these criteria but decided to apply them before looking at the data.

Children were recruited from a database of local families from a mid-sized German university city and its suburban surroundings. No data on ethnicity, socio-economic status or academic background were collected because of local data protection rules (see Singh et al., 2024 ^S1^). Caregivers provided informed consent prior to each study (when conducted in lab) or prior to general participation in our studies (when conducted in kindergarten). Children, too, gave verbal assent before starting. Families were not compensated for their participation, but children tested in the lab received a small gift (monetary value approx. $1 – $5).

**Shared features of all studies**

**Design and Material**

We applied a repeated-measures, within-subjects design with a 2 (condition) x 2 (age group) factorial design. The test condition constituted a partial ignorance task, whereas the control condition constituted a full knowledge task (both adapted from Rohwer et al., 2012^S2^). Each child participated in a total of four trials, with two subsequent trials per condition. The order of condition was counterbalanced across children (full knowledge first vs. partial ignorance first).

In all studies, we used three toy animals (bear, lion, sheep) as the main set of objects for our tasks. The animals were of similar shape and size (length: 9–11cm, height: 6–7cm) and covered in blue paint to further assimilate their outward appearance. Three additional toy animals were used in the warm-up phase (see below). In the experimental test conditions, the three main animals were hidden inside a triangular box (edge length of triangle: 55cm, height: 46cm) which consisted of one opaque side and two sides that contained either a transparent or a blurry window (window measurements: 28cm x 24cm). Note that we covered the blurry window in Studies 2 and 3 (see below, and Fig.1 in main text).

In each trial, the hidden animal was randomly selected and placed inside the box without the child and E2 knowing (blinding E2 ensured that she would not unintentionally manipulate the children’s responses). Each animal was hidden at least once; both the order of hiding events as well as which animal was hidden twice (as there were four trials but only three animals) was randomly selected.

**Procedure**

Every session started with a warm-up phase prior to the main test phase. The following paragraph describes the details of Studies 1a, 1b and 2 as they constituted the main social experiments. See the final paragraph about the non-social control Study 3.

*Warm-up.* The warm-up phase fulfilled three functions. First and foremost, the underlying objective was for E2 to establish a communicative and cooperative relationship with the participating child. Furthermore, the animals were introduced. In this first step, E1 asked the children to identify the three unambiguous animals that would later be used in test trials (bear, lion, sheep), whereas E2 was asked to identify three additional, ambiguous animals. E2 was instructed to use phrases expressing uncertainty in naming her animals. Hence, the first step of this warm-up phase aimed at i) strengthening the communicative relationship between children and E2; ii) ensuring that children could identify the three animals that would later be used in test phase; and iii) demonstrating that it was okay to acknowledge one’s own uncertainty. In a second step, once children could reliably name the three main animals, E1 introduced the three-sided box and rules of the “game” in test phase: E1 would hide one of the three animals inside the box, and the task of the person sitting in front of the opaque side was to state which animal she thought was inside. If it was the correct animal, she would be rewarded with a sticker. E2 was told to be the first one to sit in front of the opaque side, whereas the children were instructed to sit in front of either one of the windows (depending on condition). The remaining window was then covered to prevent children from peeking inside (especially relevant in the partial ignorance condition to guarantee that children remained ignorant). After pointing out that the person in front of the opaque side could not see what was hidden inside the box, E1 reminded the children that they were allowed to help E2 during the game if she needed their help.

*Test phase.* The children and E2 were instructed to close their eyes while E1 hid one of the three animals inside the box. After the hiding event, E1 told them to open their eyes, reminded E2 to say the correct animal to get a sticker, and then stepped aside to create the opportunity for the children and E2 to interact without the involvement of E1. Upon E1’s exit, E2 turned to the children for help and asked the test question (see below). If the children did not reply after the test question was repeated twice, or if they did not offer any definite advice, E2 naturally guessed any of the three animals (“Hm, okay, then I say it is a [e.g., lion]”). If they did offer advice, E2 posed a second test question (see below). E2 then called back E1 and made her final guess, always following the children’s advice. E1 opened the box and revealed the hidden animal. If the guess was correct, she rewarded E2 with a sticker; if it was false, she explained that she would not receive the reward. This procedure was repeated for the second trial. Then, E1 instructed the children to switch to the other window, and proceeded with the last two trials as before. Once the children completed all four test trials, E2 switched seats with them and now helped the children to retrieve stickers by telling them which animal was hidden inside the box. These additional trials were not analyzed as they were only conducted to assure children enjoyed the test session and felt treated fairly. In the end, E2 offered all her stickers to the children.

*Warm-up and test phase (Study 3).* The warm-up and test procedure were almost identical for the fourth (control) study. The crucial exception was that children were tested individually without the involvement of a second experimenter. Thus, E1 introduced the children to the test materials and procedures on their own, and was the one who posed the test questions throughout the experimental test phase.

**Coding**

As our main objective was children’s display of metacognitive competence, we coded whether they acknowledged their ignorance or expressed being uncertain (1) or not (0) in response to either of the two test questions in each trial. Accordingly, we expected children to score high in the two partial ignorance trials and low in the two full knowledge trials. Criteria for how strict children’s expressions were coded differed between studies (see below).

**Statistical methods**

For each study, we ran generalized linear mixed models (GLMM) with binomial error structure and logit link function to examine whether children were more likely to express uncertainty (Study 1a) or explicitly acknowledge ignorance (Studies 1b, 2 and 3) in the partial ignorance condition than in the full knowledge control condition. Following our preregistered analysis plans as closely as possible, we were interested in the fixed effects of condition, age group (and the interaction between the two in Study 1a and 1b) and order of condition, as well as the random effect of participant ID (including all random slope components of the main predictors). Please note that the order of condition is mis-specified as “trial number” in preregistrations of Study 1a and Study 1b.

As an overall test of the test predictors’ effect, we compared the full models with a respective null model lacking the test predictors but comprising the same random effect structure using a likelihood ratio test. P values for the individual effects were based on such likelihood ratio tests using R function drop1 with argument ‘test’ set to “Chisq”. All analyses were implemented in R (version 4.3.2)^S3^ using the function glmer of the R package lme4^S4^. Confidence intervals for the models were derived using the function bootMer (R package lme4), using 1,000 parametric bootstraps.

**Individual studies**

**Study 1a**

**Participants**

The final sample included a total of 66 typically developing German children aged 3 years (*n* = 34, 18 females, *age range* = 36–47 months, *mean* = 41.82, *SD* = 3.29) and 5 years (*n* = 32, 14 females, *age range* = 60–71 months, *mean* = 65.09, *SD* = 3.90). Ten additional children were tested but excluded from analyses because they neither communicated (*n* = 2) nor cooperated (*n* = 4) with the experimenters, aborted the session (*n* = 1) or because their caregivers interfered (*n* = 3). Study 1 was primarily conducted in the department’s facilities (*n* = 40), as well as in local kindergartens (*n* = 26) by two female experimenters (main: E1; and children’s partner: E2). All sessions were videotaped and coded by E1. Reliability coding by a second blinded coder for 25% of the videos revealed 100% agreement for children’s uncertainty expressions.

**Procedure and Materials**

*Warm-up*. As described above, a communicative and cooperative relationship between E2 and children was established, and children learned about the three toy animals (used for test phase) and the rules of the game. E2 identified three additional animals that were ambiguous (tiger/big cat, donkey/horse, wolf/big dog) for the sake of demonstrating that it was okay to acknowledge ignorance and uncertainty. For example, she admitted not to be sure about the identity of the wolf (“I am not sure, maybe this is a wolf? Or it could also be a dog”).

*Test phase*. The test phase was conducted as described above. In Study 1a, E2 turned to the children for help and asked the test question in a very natural and open way (“I don’t know which animal it is, but I really want to guess the correct animal so that I get a sticker. Can you help me [child’s name]? Can you tell me which animal it is?”). If children offered advice, E2 posed a follow-up question (“Ah you think it is the [animal] – are you sure?”).

**Coding.**

In Study 1a, we coded all signs of uncertainty in response to either of the two test questions as metacognitive sensitivity. This included explicit (verbal) acknowledgments of ignorance and uncertainty (e.g., “I do not know”, “I am not sure”, “maybe [animal] or maybe [animal]”), explicit (nonverbal) responses (head shaking in response to the test questions “Can you tell me which animal it is?”, “Are you sure?”), as well as implicit (verbal) uncertainty expressions (e.g., “I can tell you when I look inside”, “I could tell you from the other side”, raising voice at the end of guessing a specific animal (“the [animal]?”)) and implicit (nonverbal), behavioral responses such as information search (e.g., taking a closer look, attempting to open box or peek through transparent window), hesitation or shoulder shrugging.

**Results**

Following our preregistered steps in light of convergence issues with the full model, we removed all undefinable random slopes. The final full model that converged included the fixed effects of condition, age group, the interaction of condition and age group, and order of condition, as well as a random intercept for participant ID.

This full model (GLMM01) fitted the data significantly better compared to a null model that lacked all fixed effects but included the same random effect structure (*X^2^* = 139.17, *df* = 4, *p* < .001). However, the interaction between condition and age group was not significant (*b* = 0.77, *p* = .38). Therefore, we removed the interaction term and fitted a reduced model (GLMM02) as preregistered. Using the drop1 function, this model revealed a significant effect of condition (*b* = 4.60, *p* < .001), but no significant effects for age group (*b* = 0.34, *p* = .54) or order of condition (*b* = 0.74, *p* = .18).

**Exploratory analyses.**

Following the suggestions of four anonymous reviewers, we conducted exploratory analyses on Study 1a. First, we segregated verbal from behavioral indices of uncertainty (for those instances in which children did acknowledge uncertainty). A descriptive analysis showed that in the key partial ignorance test condition, the majority of children’s uncertainty expressions were verbal: 71.9% in 3-year-olds (41 of 57 instances) and 78.9% in 5-year-olds (45 of 57 instances). A Pearson’s chi-square test (with Yates’ continuity correction) confirmed that there was no significant association between age group and uncertainty expression (*χ²*(1, 114) = 0.43, *p* = .51): The proportion of verbal and behavioral uncertainty expressions did not differ significantly between age groups.

Secondly, we re-run our main analysis on Study 1a adopting a more conservative coding. Analogously to the analyses in Studies 1b, 2 and 3, this more conservative coding only includes children’s explicit judgments of uncertainty (explicitly referring to epistemic status, e.g. “I do not know”, “I am not sure”, “maybe” etc.). However, we would like to point out that this coding is not perfectly comparable and its meaningfulness is limited because the test questions of Study 1a did not target explicit metacognitive judgments. We fitted a binomial GLMM to predict children’s likelihood to express uncertainty explicitly by condition, age group, the interaction of condition and age group, the order of condition and individual variation (including random intercepts for each child). The model revealed a significant effect of condition (*b* = 4.82, *p* < .001), but no other significant effects (all *p*s > .15). Thus, we followed our original analysis plan and fitted a reduced model that excluded the interaction term between condition and age group. This reduced model confirmed a significant effect of condition (*b* = 5.5, *p* < .001) and found no significant effects of age group (*b* = 1.15, *p* =.17) or order of condition (*b* = -0.38, *p* = .65). This shows that the original finding of Study 1a holds even with a more conservative approach to coding: Both 3- and 5-year-old children provided explicit metacognitive judgments and explicitly acknowledged their own uncertainty significantly more in the crucial partial ignorance condition. Thus, Study 1a robustly shows metacognitive competence in our social version of the partial ignorance task.

**Study 1b**

**Participants**

The final sample included a total of 64 typically developing German children aged 3 years (*n* = 32, 17 females, *age range* = 36–47 months, *mean* = 42.13 months, *SD* = 3.17) and 5 years (*n* = 32, 15 females, *age range* = 60–71 months, *mean* = 66.41 months, *SD* = 3.04). Five additional children were tested but excluded from analyses because they did not communicate with the experimenters (*n* = 1), did not complete the session (*n* = 1), cheated in the crucial test condition (*n* = 1; categorized as experimental error since experimenters could not prevent the child’s cheating), or because of parental interference (*n* = 2). All participants were tested in the department’s facilities by two female experimenters (E1 and E2). All sessions were videotaped and coded by one of the experimenters. Reliability coding by a second blinded coder for 25% of the videos revealed 100% agreement for children’s ignorance acknowledgements.

**Procedure and Materials**

*Warm-up*. We used the same materials and procedures as in Study 1a, except that E2 had to identify two difficult animals (some subspecies of dinosaurs and antelopes) in addition to one of the ambiguous animals (donkey/horse). When identifying the two difficult animals during warm-up, E2 included explicit expressions of ignorance (e.g., “I do not know what this is supposed to be”) so that children learned that it was okay to acknowledge one’s own ignorance.

*Test phase*. The test phase was conducted as described above. However, in crucial difference to Study 1a, the test questions were rephrased to explicitly refer to children’s epistemic state and avoid ambiguous responses (as E2 combined two questions in Study 1a “Can you help me? Can you tell me (...)?” which made interpretation of children’s answers difficult): When E2 turned to the children for help, she asked a single question (“I don’t know which animal it is, but I really want to say the correct animal so that I get a sticker. Do you know which animal it is?”) and, if necessary, followed up to obtain a definite metacognitive answer (“And do you know this?”). These modifications reduced the vagueness and avoided ambiguous responses resulting from the more natural and double-barreled question used in Study 1a. Moreover, E2 now avoided all “guessing” vocabulary (both in the key test question and when making her own guess) to minimize the possibility that children would misunderstand the partial ignorance condition as a guessing game and feel prompted to guess, too.

**Coding.**

As we coded any kind of uncertainty in Study 1a, one could argue that this resulted in too liberal coding and thus, overestimation of children’s metacognitive insight. To this end, children only received an ignorance score (1) if they explicitly negated the main test question (e.g., “no, I do not know”, head shaking), or if they acknowledged their initial response being a guess by negating the follow-up question (e.g., “maybe the lion” – “do you know this?” – “no”). Note that nonverbal negation responses like head shaking were indicative of explicit ignorance acknowledgments, as well. In contrast, implicit markers of children’s uncertainty, such as shoulder shrugging or information-seeking behavior, were not coded as acknowledging ignorance (0).

**Results**

As in Study 1a, we followed our preregistered procedure for fitting our full model. The full model that eventually converged included the fixed effects of condition, age group, the interaction of condition and age group, and order of condition, as well as a random intercept for participant ID.

This full model (GLMM03) fitted the data significantly better compared to a null model that lacked all fixed effects but included the same random effect structure (*X^2^* = 165.68, *df* = 4, *p* < .001). Because the interaction between condition and age group was significant (*b* = -2.89, *p =* .02), we ran separate analyses of the effect of condition for both age groups (as preregistered).

Model comparison of the 3-year-olds’ full and respective null model revealed that the full model (GLMM04) fitted the data significantly better (*X^2^* = 104.27, *df* = 2, *p* < .001). This full model revealed a significant effect of condition (*b* = 7.02,*p* < .001) but no significant effect of order of condition (*b* = -1.27, *p* = .15), meaning that 3-year-old children explicitly acknowledged their ignorance significantly more often under partial ignorance than when fully knowledgeable, independently of which condition was presented first.

Model comparison of the 5-year-olds’ full and respective null model revealed that the full model (GLMM05) fitted the data significantly better (*X^2^* = 60.08, *df* = 2, *p* < .001). Here, the full model revealed a significant effect of condition (*b* = 4.86, *p* < .001) as well as a significant effect of order of condition (*b* = -1.85, *p* = .04). 5-year-old children also acknowledged their ignorance significantly more often in the partial ignorance than full knowledge condition, and more so when they experienced the full knowledge condition first.

**Study 2**

**Participants**

The final sample included a total of 32 typically developing German 3-year-old children (*n* = 32, 15 females, *age range* = 36–47 months, *mean* = 41.62 months, *SD* = 2.77). Study 2 was conducted to control for the possibility that children performed better in our task just because the blurry window served as a visual reminder of their uncertainty. As we assumed that the blurry window would affect performance in both age groups equally, and since we were interested in testing whether *the earliest* forms of explicit metacognition are boosted by social contexts, we decided to test only the younger age group. Seven additional children were tested but excluded from analyses because they did not communicate with the experimenters (*n* = 1), did not complete the session (*n* = 4), or had language difficulties (*n* = 2). Again, all participants were tested in the department’s facilities by two female experimenters (E1 and E2). All sessions were videotaped and coded by one of the experimenters. Reliability coding by a second blinded coder for 36% of the videos revealed 100% agreement for children’s ignorance acknowledgments.

**Procedure and Materials**

We followed the exact same procedure as in Study 1b. The only difference was that the partial ignorance condition took place in front of a completely opaque side instead of a blurry window.

**Coding**

We applied the same conservative coding scheme as in Study 1b.

**Results**

Again, we followed our preregistered procedure in fitting the full model. The converging model included only condition and the order of condition as fixed effects and random slopes for individual participants. This full model (GLMM06) fitted the data significantly better compared to a null model that lacked all fixed effects but included the same random effect structure (*X^2^* = 73.39, *df* = 2, *p* < .001). With this model, a significant effect of condition (*b* = 7.47, *p* < .001) was revealed, and a significant effect of order of condition (*b* = -2.49, *p* = .04). 3-year-old children explicitly acknowledged their ignorance significantly more under partial ignorance than under full knowledge.

We conducted exploratory analyses across the 3-year-old children from both Studies 1b and 2 and fitted a model which included the fixed effects of condition, study ID, order of conditions and random intercept for participant ID. The full-null model comparison revealed that the full model (GLMM07) fitted the data significantly better than the null model (*X^2^* = 177.71, *df* = 3, *p* < .001). Condition was a significant predictor of 3-year-olds’ ignorance statements (*b* = 7.55, *p* < .001) as well as the order of condition (*b* = -1.86, *p* = .02), but study ID was not (*b* = -1.23, *p* = .10). This means that 3-year-olds’ metacognitive performance in the partial ignorance condition was not significantly different between Study 1b (in front of a blurry window) and Study 2 (without a window at all).

**Study 3**

**Participants**

The final sample included a total of 33 typically developing German 3-year-old children (*n* = 33, 16 females, *age range* = 36–47 months, *mean* = 40.24 months, *SD* = 3.28). One additional child was tested but no analyzable data were obtained due to language difficulties (*n* = 1). Study 3 aimed at providing a direct comparison of our social partial ignorance task (Studies 1b and 2) with a non-social control group. Thus, Study 3 was conducted with only one experimenter (the same E1) in local kindergartens. All sessions were videotaped and coded by E1; reliability coding for 25% of the videos revealed 100% agreement with an independent coder.

**Procedure and Materials**

To control for the alternative explanation that factors other than the social manipulation scaffolded children’s metacognitive performance, Study 3 provided a non-social condition, closely matched to the social conditions in Studies 1a, 1b and 2. Hence, children were tested by the same main experimenter (E1) with the same materials and experimental setup as in Study 2 – the crucial difference being the absence of a cooperative partner and thus the social context. E1 explained that children were playing on their own, and after hiding the toy animals inside the box, she directly posed the metacognitive test questions.

**Coding**

We applied the same conservative coding scheme as in Studies 1b and 2.

**Results**

Again, we followed our preregistered procedure in fitting the full model. The converging model included only condition and the order of condition as fixed effects and random slopes for individual participants. This full model (GLMM08) fitted the data significantly better compared to a null model that lacked all fixed effects but included the same random effect structure (*X^2^* = 40.72, *df* = 2, *p* < .001). With this model, a significant effect of condition (*b* = 4.32, *p* < .001) was revealed, but no significant effect of order of condition (*b* = 0.77, *p* = .43). Three-year-old children in the non-social version of our task acknowledged their ignorance significantly more under partial ignorance than under full knowledge.

We conducted exploratory analyses across the 3-year-old children from Studies 2 and 3 to directly compare the studies most closely matched (in a minimal contrast) in all respects except the social vs. individual task format. Hence, we fitted a model including the fixed effects of condition, study ID, their interaction, order of condition and random intercept for participant ID. The full-null model comparison revealed that the full model (GLMM09) fitted the data significantly better than the null model (*X^2^* = 112.84, *df* = 4, *p* < .001). Importantly, this full model did not reveal a significant interaction between study and condition (*b* = -2.22, *p* = .11) and no significant effect for order of condition (*b* = -0.68, *p* = .38), indicating that the effect of condition tended to be stronger in Study 3, i.e., in the social compared to the non-social task.

**Exploratory analyses across studies**

As discussed in the main text, we conducted exploratory analyses across Studies 1b, 2, and 3 to control for the possibility that children’s performance does not reflect their metacognitive insight but that they interpreted repeated metacognitive test questions as an indicator that their previous answers were wrong. To control for such pragmatic artifacts, we analyzed children’s answer patterns of when they eventually gave competent answers in a partial ignorance trial (“Don’t know”). Did they acknowledge their ignorance spontaneously (already in response to the first test question “Do you know which animal is in the box?”) or only after repeated questioning (e.g., “It is the lion”; but when then asked “The lion - Do you know this?” they would switch to saying “No”)?

Thus, for Studies 1b, 2, and 3, we coded whether children initially negated the first test question or whether they eventually negated the second test question after initially providing an affirmative answer (“Yes, I know”) or guess (“Bear”). Remarkably, the majority of 3-year-olds across contexts spontaneously admitted that they did not know which animal was hidden inside the box. In the social context (Studies 1b and 2), around 90% of competent children spontaneously acknowledged their ignorance (*n* = 45 partial ignorance trials and *n* = 36 partial ignorance trials in Study 1b and 2, respectively); only few children initially guessed an animal but then revised their answer (Study 1b: *n* = 4 partial ignorance trials; Study 2: *n* = 1 partial ignorance trials) and even fewer admitted to their ignorance after initially claiming to know (Study 1b: *n* = 1 partial ignorance trials; Study 2: *n* = 2 partial ignorance trials). As revealed by Fisher’s Exact Test, this pattern was not significantly different for competent children in the non-social context (Study 3, *p* = .09), where around 70% of competent ignorance statements were made spontaneously (*n* = 21 partial ignorance trials).

**Elaboration on ethical approval of our studies**

This research was conducted in accordance with the Declaration of Helsinki and the Ethical Principles of the German Psychological Society (DGPs), the Association of German Professional Psychologists (BDP), and the American Psychological Association (APA). It involved no invasive or otherwise ethically problematic techniques and no deception (and therefore, according to National jurisdiction, did not require a separate vote by a local Institutional Review Board; see the regulations on freedom of research in the German Constitution (§ 5 (3)), and the German University Law (§ 22)).

**SI References**

S1	Singh, L., Barokova, M. D., Baumgartner, H. A., Lopera-Perez, D. C., Omane, P. O., Sheskin,
M., Yuen, F. L., Wu, Y., Alcock, K. J., Altmann, E. C., Bazhydai, M., Carstensen, A., Chan, K. C. J., Chuan-Peng, H., Dal Ben, R., Franchin, L., Kosie, J. E., Lew-Williams, C., Okocha, A., … Frank, M. C. (2024). A unified approach to demographic data collection for research with young children across diverse cultures*. Developmental Psychology, 60*(2), 211–227. https://doi.org/10.1037/dev0001623

S2	Rohwer, M., Kloo, D., & Perner, J. (2012). Escape from metaignorance: How children develop	an understanding of their own lack of knowledge. *Child development, 83*(6), 1869–1883.

S3	R Core Team (2023). R: A Language and Environment for Statistical Computing. *R	Foundation for Statistical Computing*, Vienna, Austria.

S4	Bates, D., Mächler, M., Bolker, B., and Walker, S. (2015). Fitting linear mixed-effects models	using lme4. *Journal of Statistical Software, 67*(1):1–48.
